# Peptides as multifunctional players in cancer therapy

**DOI:** 10.1038/s12276-023-01016-x

**Published:** 2023-06-01

**Authors:** Sri Murugan Poongkavithai Vadevoo, Smriti Gurung, Hyun-Su Lee, Gowri Rangaswamy Gunassekaran, Seok-Min Lee, Jae-Won Yoon, Yun-Ki Lee, Byungheon Lee

**Affiliations:** 1 Department of Biochemistry and Cell Biology, Kyungpook National University, 680 Gukchaebosang-ro, Jung-gu, Daegu, 41944 Republic of Korea; 2 Department of Biomedical Science, Kyungpook National University, 680 Gukchaebosang-ro, Jung-gu, Daegu, 41944 Republic of Korea; 3 Cell & Matrix Research Institute, School of Medicine, Kyungpook National University, 680 Gukchaebosang-ro, Jung-gu, Daegu, 41944 Republic of Korea; 4 Department of Physiology, Daegu Catholic University School of Medicine, 33 Duryugongwon-ro 17-gil, Nam-gu, Daegu, 42472 Republic of Korea

**Keywords:** Targeted therapies, Drug delivery, Targeted therapies

## Abstract

Peptides exhibit lower affinity and a shorter half-life in the body than antibodies. Conversely, peptides demonstrate higher efficiency in tissue penetration and cell internalization than antibodies. Regardless of the pros and cons of peptides, they have been used as tumor-homing ligands for delivering carriers (such as nanoparticles, extracellular vesicles, and cells) and cargoes (such as cytotoxic peptides and radioisotopes) to tumors. Additionally, tumor-homing peptides have been conjugated with cargoes such as small-molecule or chemotherapeutic drugs via linkers to synthesize peptide–drug conjugates. In addition, peptides selectively bind to cell surface receptors and proteins, such as immune checkpoints, receptor kinases, and hormone receptors, subsequently blocking their biological activity or serving as hormone analogs. Furthermore, peptides internalized into cells bind to intracellular proteins and interfere with protein–protein interactions. Thus, peptides demonstrate great application potential as multifunctional players in cancer therapy.

## Cancer treatment: peptides offer options for targeted therapeutic delivery

Targeted drug delivery systems using tiny, non-toxic guiding molecules could transform cancer treatment and reduce chemotherapy resistance. Peptides are small molecules consisting of two or more amino acids. While they degrade quickly in the body, they show promise as guiding molecules for targeted drug delivery, with deep tissue penetration and low toxicity. Byungheon Lee at Kyungpook National University in Daegu, South Korea, and co-workers reviewed progress into novel therapeutic systems that use ‘tumour-homing’ peptides to target solid tumours. Adding peptides to nanoparticles that carry chemotherapy drugs enhances their penetration and accumulation in target tumours. Peptide-assisted delivery can also reduce the major adverse effects of chemotherapy, and appear to be widely tolerated in the body, even in the brain. Peptides that bind to and inhibit surface receptor proteins on tumour cells could also block cancer progression.

## Introduction

**Fig. 1:**
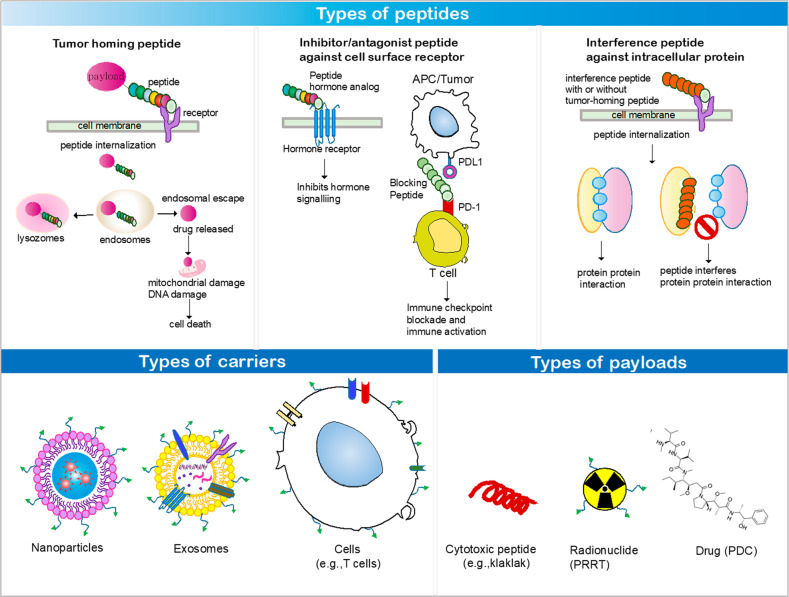
Types of peptides, carriers and payloads. Tumor-homing peptides bind to their receptors on tumor cells and selectively deliver cargoes therein, causing cell damage and death. Antagonist peptides target cell surface receptors on tumor cells, such as hormone receptor and PD-L1, and inhibit their biological activities. Interference peptides with or without tumor-homing peptides enter cells, bind to their intracellular targets, and inhibit the interaction between the target and its binding partner. Various types of carriers, such as nanoparticles, exosomes and cells, and cargoes, such as cytotoxic peptides, radionuclides, and drugs (PDCs), are used for targeted delivery via tumor-homing peptides.

Compared with antibodies, peptides exhibit certain disadvantages, such as lower affinity, rapid excretion from the body (or shorter half-life in the body), and vulnerability to protease-mediated degradation. Conversely, the advantages of peptides include deep tissue penetration, efficient internalization into cells, lower immunogenicity and toxicity to the bone marrow and liver, and easy modification via chemicals compared with antibodies^1–4^. The pros and cons of peptides relative to antibodies are summarized in Table 1. Presently, >80 peptide therapeutics are available on the market; these include liraglutide (Victoza®), a glucagon-like peptide-1 used to treat type 2 diabetes mellitus, and leuprolide (Lupron®), a somatostatin analog used to treat prostate cancer^4^. In addition, peptides have been employed to identify peptide mimotopes^5^, generate vaccines^6^, and map protein–protein interaction epitopes^7^. Herein, we focused on the multifunctional application of peptides in targeted therapeutics; peptides can deliver carriers (such as nanoparticles, extracellular vesicles (EVs), and cells) and cargoes (such as cytotoxic peptides, radioisotopes, and small molecules) to target cells; inhibit or antagonize cell surface receptors and proteins; and interfere with intracellular protein–protein interactions. The various peptides, carriers, and cargoes described herein are summarized in Fig. 1.

**Table 1:** Comparison between peptides and antibodies as tumor-homing ligands.

	Peptide	Antibody
Affinity	Lower (nM–μM)	Higher (pM–nM)
Stability	More vulnerable to degradation	Less vulnerable to degradation
Body clearance and half-life in the blood	Faster clearance and shorter half-life (~hours)	Slower clearance and longer half-life (~3 weeks)
Target tissue accumulation	Faster	Slower
Size (molecular weight)	Smaller (1–3 kDa)	Larger (150 kDa)
Tissue penetration	Deeper and faster	Perivascular and slower
Internalization into cells	More efficient	Less efficient
Controlled chemical modification	Easier	More difficult
Immunogenicity and toxicity (liver, bone marrow)	Lower	Higher
Production, quality control (QC), and cost	Chemical synthesis, easier QC, and lower cost	Cell culture or animal, more difficult QC, and higher cost

## Tumor-homing peptides as targeting ligands

### Peptide-targeted delivery of nanoparticles

Tumor-homing peptides have been used for guiding nanoparticles to cancer cells through direct interactions between the peptides and receptors or binding partners on the cell surface^8^. In general, they are designed to be tumor cell-specific to enhance the internalization of nanoparticles into tumor cells. Multivalent labeling of peptides on nanoparticles increases the binding avidity of the peptide. In addition, the conjugation of peptides with nanoparticles tends to protect the peptide from protease-mediated degradation. The most well-known tumor-homing peptide is the RGD peptide, including RGD4C (ACDCRGDCFCG) and Cilengitide™ (RGDfV), which bind to overexpressed αvβ3 integrin in the angiogenic endothelial cells of tumor blood vessels, thereby inhibiting angiogenesis^9–11^. The conjugation of the RGD peptide with drugs or drug-loaded nanoparticles has been intensively investigated for cancer therapy^12^. Internalized RGD or iRGD (CRGDR/KGPDC), a modified version of the RGD peptide, not only bound to αV integrins but also increased the tissue penetration of drugs. The binding of the RGD motifs to the αV integrins expressed in tumor endothelial cells induces the protease-mediated cleavage of the iRGD peptide, producing two peptides, namely, CRGDR/K and GPDC. Subsequently, the CRGDR/K peptide containing the C-terminal CendR motif (R/KXXR/K) binds to neuropilin-1, activating an endocytic pathway^13–15^. Thus, iRGD increases the tissue penetration of drugs regardless of whether it is conjugated to or coadministered with the drug^16–18^.

The mitochondrial protein p32 or gC1qR is overexpressed in tumors with aberrant cell surface expression in tumor cells, tumor lymphatics, and a subset of myeloid cells such tumor-associated macrophages (TAMs)^19^. When conjugated with the p32-binding LyP-1 peptide (CGQKRTRGC), Abraxane, a nanoparticle albumin-bound paclitaxel, accumulated in tumor tissues and inhibited tumor growth more efficiently than untargeted Abraxane^19,20^. Vascular endothelial growth factor receptor 2 (VEGFR-2) is predominantly expressed on the surface of tumor endothelial cells^21^. Paclitaxel-loaded nanoparticles conjugated with K237 peptide (HTMYYHHYQHHL), a VEGFR-2-binding peptide, efficiently inhibited angiogenic activity and induced apoptosis of tumor endothelial cells and necrosis of tumor tissues^22^. Interleukin-4 receptor (IL4R), particularly type-II IL4R, is composed of IL4Rα and IL13Rα1, and it is upregulated in major tumors such as breast, lung, head and neck tumors and glioblastoma compared to their corresponding control tissues^23–25^. IL4RPep-1 peptide (CRKRLDRNC), an IL4R-binding peptide, can enhance the delivery of nanoparticles to IL4R-overexpressing tumors^26–30^. In addition, IL4R is highly expressed in M2-polarized, protumoral TAMs compared with M1-polarized, antitumoral macrophages, making IL4R a potential target for targeted drug delivery to TAMs^31,32^. The mannose receptor CD206 is also considered a cell surface marker of M2-type macrophages^33^. Nanoparticles labeled with a mUNO peptide (CSPGAK), which is a CD206-binding peptide, promote selective drug delivery to M2-type TAMs and induce M2 to M1 reprogramming of the macrophage phenotype^33^. The tumor-homing peptides used for the delivery of nanoparticles are summarized in Table 2.

**Table 2:** Tumor-homing peptides used for guided delivery of nanoparticles to tumors.

Name	Sequence	Target	Target disease	Reference
RGD4C	ACDCRGDCFCG	Integrin αvβ3	Melanoma, colon tumor ovarian tumor glioblastoma	^9–11^
iRGD	CRGDR/KGPDC	Integrin αvβ3	Glioblastoma, melanoma	^16–18^
LyP-1	CGQKRTRGC	P32	Melanoma	^20^
K237	HTMYYHHYQHHL	VEGFR-2	Breast tumor	^22^
IL4RPep-1	CRKRLDRNC	IL4R	Lung tumor, breast tumor, colon tumor	^26–30^
mUNO	CSPGAK	CD206	Breast tumor	^33^

### Peptide-targeted delivery of EVs or exosomes

EVs or exosomes are endogenous nanoparticles secreted from cells into circulation. They can carry DNA, RNA, proteins, and lipids and distribute them among cells. Labeling tumor-homing peptides on the surface of exosomes loaded with therapeutics can reduce major adverse side effects in cancer therapy^34^. The surface modification of exosomes is performed via two methods: genetic and nongenetic engineering. Using genetic engineering, dendritic cells (DCs) have been engineered to secrete exosomes expressing Lamp2, an exosomal membrane protein, fused with the neuron-specific RVG peptide (YTIWMPENPRPGTPCDIFTNSRGKRASNG)^35^. Subsequently, RVG peptide-guided exosomes were employed to deliver short interfering RNA to neurons, microglia, and oligodendrocytes in the brain by targeting the gamma-aminobutyric acid (GABA) receptor, inducing target gene knockdown with negligible nonspecific uptake in other tissues^35^. Similarly, mouse immature DCs were genetically engineered to secrete exosomes expressing the Lamp2 protein fused with the iRGD peptide (CRGDR/KGPDC) and demonstrated highly efficient targeted drug delivery to αv integrin-positive breast cancer cells, consequently inhibiting tumor growth^36^. Cellular exosomes engineered to express the transmembrane domain of platelet-derived growth factor receptor fused with the GE11 peptide (YHWYGYTPQNVI), an epidermal growth factor receptor (EGFR)-binding peptide, selectively delivered let-7a microRNA to breast cancer tissues^37^. Moreover, tumor cell-derived exosomes genetically engineered to express a pH-sensitive fusogenic GALA peptide (WEAALAEALAEALAEHLAEALAEALEALAA) efficiently delivered tumor antigens to the cytoplasm of DCs and promoted the tumor antigen presentation of DCs via the major histocompatibility complex class I molecule^38^.

Exosomal surfaces have also been nongenetically modified using lipid-based membrane anchors, electrostatic interactions, and ligand–receptor interactions. M1 macrophage-derived exosomes were transfected with NF-κB p50 siRNA and miR-511-3p to foster M1 polarization and subjected to surface modification with the IL4R-targeting IL4RPep-1 peptide (CRKRLDRNC) using a phospholipid anchor; these constructs inhibited tumor progression by reprogramming IL4R-high and M2-polarized TAMs to an M1-like phenotype^39^. The surface modification of blood exosomes with transferrin-conjugated superparamagnetic nanoparticles via interaction with the transferrin receptor, with L17E endosomolytic peptides (IWLTALKFLGKHAAKHEAKQQLSKL) via electrostatic interactions, and with cholesterol-conjugated miR-21 inhibitor by anchoring to the lipid membrane increased tumor accumulation and drug delivery and enabled efficient endosomal escape^40^. Exosome surface labeling with a chimeric peptide (C16K-protoporphyrin IX-PKKKRKV) comprising an alkyl chain (C16), photosensitizer (protoporphyrin IX), and nuclear localization signal peptide (PKKKRKV) can enhance the nuclear delivery of the photosensitizer and efficiently inhibit tumor growth via photodynamic therapy^41^. The tumor-homing peptides used for the delivery of EVs are summarized in Table 3.

**Table 3:** Tumor-homing peptides used for guided delivery of extracellular vesicles or exosomes.

Name	Sequence	Target	Target disease	Reference
RVG	YTIWMPENPRPGTPCDIFTNSRGKRASNG	GABA receptor	Brain disease	^35^
iRGD	CRGDR/KGPDC	Integrin αvβ3	Breast tumor	^36^
GE11	YHWYGYTPQNVI	EGFR	Breast tumor	^37^
GALA	WEAALAEALAEALAEHLAEALAEALEALAA	Acidic pH	melanoma	^38^
IL4RPep-1	CRKRLDRNC	IL4R	Breast tumor Lung tumor	^39^
L17E	IWLTALKFLGKHAAKHEAKQQLSKL	Endosomal membrane	Glioblastoma Breast tumor	^40^

### Peptide-guided delivery of cells

Enhancing the tumor homing of cytotoxic T lymphocytes (CTLs) in adoptive cell therapy is of high demand. Thus, chimeric antigen receptor (CAR)-T cells have been used to address this limitation. CAR-T cells are genetically engineered to express a chimeric receptor composed of an antibody against a tumor antigen (such as CD19), a cytoplasmic domain of the zeta chain of the T-cell receptor, and a costimulator domain^42,43^. In contrast, the nongenetic modification of the cell surface can reduce unexpected risks caused by genetic engineering of cells. CTLs labeled with the IL4R-binding IL4RPep-1 peptide (CRKRLDRNC) using a phospholipid-based membrane anchor showed enhanced tumor homing and antitumor growth activity in mice bearing B16F10 melanoma^44^. Apart from CTLs, mesenchymal stem cells (MSCs) conjugated with an E-selectin-targeting peptide (CGSDITWDQLWDLMK) on the cell surface showed controlled adhesion and rolling through an interaction between the peptide on the stem cells and E-selectin on the endothelial cells^45^. In addition, the nongenetic surface modification of MSCs with sialyl Lewis^X^ carbohydrate using a polyacrylamide linker and biotin/streptavidin interaction showed robust rolling on the endothelium and homed inflamed tissues in vivo more efficiently than unlabeled MSCs^46^.

### Peptide-targeted cytotoxic peptides

Cationic amphipathic peptides with inherent cytotoxicity exhibit advantages: they can attenuate multidrug resistance in tumor cells and present broad-spectrum antitumor activities^47,48^. In contrast, they have drawbacks, including poor membrane permeability, suboptimal therapeutic activity, and structural instability^48^. A typical example is the KLAKLAKKLAKLAK or (KLAKLAK)2 proapoptotic peptide, which was originally developed as an antimicrobial peptide. In mammalian cells, it triggers mitochondrial membrane disruption and cytochrome C release, subsequently inducing cell apoptosis^49,50^. The (KLAKLAK)2 peptide encapsulated into mesoporous nanoparticles induced mitochondrial swelling and apoptosis^51^. Combining the (KLAKLAK)2 peptide with the CNGRC peptide, an aminopeptidase N-targeting peptide, efficiently inhibited tumor growth by targeting the enzyme present in angiogenic tumor endothelial cells^52^. The (KLAKLAK)2 peptide fused with the IL4R-binding IL4RPep-1 peptide (CRKRLDRNC) exhibited selective cytotoxicity toward IL4R-expressing tumor cells and enhanced the sensitivity of cells to chemotherapy^53^. The IL4R-targeted (KLAKLAK)2 peptide acted on IL-4R-high and M2-polarized TAMs as well as tumor cells and reduced the proportion of M2-type TAMs in the tumor microenvironment^54^. Moreover, the (KLAKLAK)2 peptide guided by CD44v6-binding (CNLNTIDTC and CNEWQLKSC), Her-2-binding (YCDGFYACYMDV), prostate tumor-targeting (SMSIARL), and bladder tumor-targeting (CSNRDARRC) peptides efficiently inhibited tumor growth with minimal effects on normal tissues^55–59^.

In addition, other cytotoxic or lytic peptides, such as defensin 1 (ACYCRIPACIAGERRYGTCIYQGRLWAFCC), cecropin B (KWKVFKKIEKMGRNIRNGIVKAGPAIAVLGEAKAL), magainins (GIGKFLHSAKKFGKAFVGEIMSNS), and dermaseptin (ALWKEVLKNAGKAALNEINNLVG), can increase membrane permeability and promote cell death^60,61^. The lactoferrin 5 derivative (PAWRKAFRWAWRMLKKAA) also showed selective cytotoxicity to tumor cells^62^. The eMTD peptide (KLNFRQKLLNLISKLFCSGT), consisting of the BH3 domain and mitochondrial targeting domain of the Noxa protein, causes cell membrane damage and necrotic cell death by interacting with voltage-dependent anion channel 2^63^. Moreover, a peptide consisting of a prostate-specific membrane antigen (PSMA) substrate linked to a membrane-disrupting amoebapore H3 peptide (GFIATLCTKVLDFGIDKLQLIEDK) was highly active against PSMA-expressing LNCaP prostate cancer cells but not against PSMA-negative PC3 prostate cancer cells^64^. The cytotoxic peptides described in this section are summarized in Table 4.

**Table 4:** Cytotoxic peptides.

Name	Sequence	Target disease/cell type	Reference
(KLAKLAK)2	KLAKLAKKLAKLAK	Breast cancer, lung cancer, bladder cancer	^52–58^
Defensin 1	ACYCRIPACIAGERRYGTCIYQGRLWAFCC	Skin diseases, breast cancer, lung cancer, Prostate cancer	^60,61^
Cecropin B	KWKVFKKIEKMGRNIRNGIVKAGP AIAVLGEAKAL	Skin diseases, breast cancer, lung cancer, Prostate cancer	^60,61^
Magainin	GIGKFLHSAKKFGKAFVGEIMSNS	Skin diseases, breast cancer, lung cancer, Prostate cancer	^60,61^
Dermaseptin	ALWKEVLKNAGKAALNEINNLVG	Skin diseases, breast cancer, lung cancer, Prostate cancer	^60,61^
Lactoferrin 5	PAWRKAFRWAWRMLKKAA	Sarcoma, leukemia, colorectal cancer	^62^
eMTD	KLNFRGKLLNLISKLFCSGT	HeLa cervical cancer cells	^63^
H-3	GFIATLCTKVLDFGIDKLQLIEDK	Prostate cancer	^64^

### Peptide-targeted radionuclides: Peptide receptor radionuclide therapy

Peptide receptor radionuclide therapy (PRRT) involves the combination of a tumor-homing peptide with a radionuclide or radioactive isotope as the therapeutic substance. The advantages of PRRT include its selectiveness in delivering radionuclides, which reduces systemic side effects, and its effective control of advanced, inoperable or metastatic tumors; however, radiation-induced toxicity to healthy organs, especially the bone marrow, remains a major limitation^65^. Octreotide (Sandostatin®, FCFWKTCT), an 8-mer peptide of somatostatin analog, plays a vital role in treating patients with neuroendocrine tumors^66^. PRRT with octreotide aims to selectively irradiate somatostatin receptor 2 (SSTR2)-expressing neuroendocrine tumor cells and the surrounding blood vessels to inhibit the angiogenetic response during treatment^67^. ^111^In is linked to octreotide using diethylenetriamine pentaacetic acid, while ^90^Y and ^177^Lu (Lutathera®) are linked using tetraazacyclododecane tetraacetic acid as a chelator^68^. In addition to SSTR2, PRRT has been extended to other receptors, such as the gastrin-releasing peptide (GRP) and cholecystokinin-2 (CCK-2) receptors. ^99m^Tc-conjugated RP527 peptide (VPLPAGGGTVLTKMYPRGNHWAVGHLM), a GRP analog, has been exploited for treating human malignancies, including colon and prostate carcinomas^69^. ^111^In-labeled minigastrin (LEEEEEAYGWMDF), a CCK-2 receptor-selective peptide, has been employed to treat human colorectal and pancreatic tumors^70^.

### Peptide-targeted small-molecule drugs: peptide–drug conjugates

**Fig. 2:**
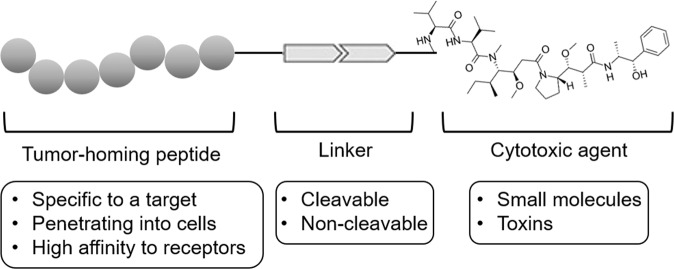
Structure of peptide–drug conjugates (PDCs). PDCs comprise a tumor-homing peptide, linker, and cytotoxic agent. The linkers used for PDCs are cleaved by intracellular enzymes or the acidic pH environment inside tumor cells, whereas some linkers are noncleavable. Small molecules are commonly used as cytotoxic agents for PDCs, and in certain cases, bacterial toxins are used.

Peptide–drug conjugates (PDCs) comprise three elements: a tumor-homing peptide, linker, and cytotoxic agent (Fig. 2). Small molecule-based cytotoxic agents have advantages of high oral availability, metabolic stability, and high membrane permeability, while having disadvantages of high toxicity, poor solubility, and lower selectivity than alternatives^71^. The delivery of PDCs into tumor cells via tumor-homing peptides can exert a tumoricidal effect in the intracellular compartments of tumor cells where tumor-specific pH or enzymes can break the linkers, releasing the drugs. Considering that PDCs increased the local concentration of cytotoxic agents in tumor tissues, they can reduce cytotoxic effects to normal tissues and increase therapeutic efficacy. For antibody–drug conjugates (ADCs), the market size in terms of revenue is predicted to exceed 16 billion dollars by 2026^72^. Compared with ADCs, PDCs exhibit better tumor penetration because of their small molecular weight, lower systemic exposure (owing to rapid clearance from the body), lower risk of immunogenicity and liver damage, and easier and cheaper production methods.

Diverse linkers have been designed to conjugate drugs or cytotoxic agents with tumor-homing peptides^73,74^. Selecting an appropriate linker is crucial for designing PDCs. Furthermore, the microenvironment where PDCs function should be considered because linkers impact drug efficacy or binding affinity depending on structural differences of the linkers. For example, certain types of peptide linkers are designed to be cleaved by enzymes abundant in tumor cells to selectively release drugs to these cells. These linkers include the GFLG peptide which is cleaved by cathepsin B^75^, the PLGLAG peptide which is cleaved by matrix metalloprotease (MMP)-2/9^76^, and the oxime-hydrazone bond which is hydrolyzed in an acidic pH^77^.

The SSTR2-binding octreotide was conjugated to doxorubicin via a cleavable disulfide bond and used for the treatment of pituitary, pancreatic, and breast tumors^78^. The disulfide bonds can be cleaved by reduced glutathione (GSH) in cells. The αvβ3 integrin-binding RGD4C peptide was conjugated to PD0325901, an MEK1/2 inhibitor, via a GGGGG peptide linker, which enhanced the antitumor activity of the drug against glioblastoma^79^. The RGDfK peptide-camptothecin conjugate linked by a Lys splitter enhanced cytotoxicity to melanoma and non-small cell lung cancer cells^80^. EGFR-binding GE11 peptide was linked to doxorubicin via the disulfide bond and used for hepatocellular tumors^81^. The angiopep-2 peptide that binds to low-density lipoprotein receptor-related protein-1 (LRP-1) was conjugated to paclitaxel via a succinyl group (named ANG1005) and applied to the treatment of glioma and metastatic breast cancer^82^. The tumor-homing peptides and linkers involved in the generation of PDCs are summarized in Table 5. Several PDCs are being considered for approval by the Food and Drug Administration (FDA) for commercial use. For example, BT8009, comprising a bicyclic peptide (CP(1Nal)dCM(hArg)DWSTP(HyP)WC) as a targeting moiety and monomethyl auristatin (MMAE) as a cargo, targets Nectin-4 on tumor cells. This PDC is in phase I/II clinical trials for the treatment of patients with metastatic non-small cell lung cancer. The PDCs currently under clinical/preclinical trials are summarized in Table 6.

**Table 5:** Peptides and linkers used for generating peptide–drug conjugates.

Peptide name	Sequence	Target	Linker	Target disease	Reference
Octreotide	FCFWKTCT	SSTR2/5	disulfide	Pituitary tumor, pancreatic cancer, breast tumor	^78^
RGD4C	ACDCRGDCFCG	Integrin αvβ3	GGGGG	Glioblastoma, Kaposi’s Sarcoma	^12,79^
RGDfK	cyclic, RGDfK	Integrin αvβ3	Lys splitter	Melanoma, non-small cell lung cancer	^80^
GE11	YHWYGYTPQNVI	EGFR	disulfide	Hepatocellular carcinoma	^81^
Angiopep-2	TFFYGGSRGKRNNFKTEEY	LRP-1	Succinyl group	Glioma, metastatic breast cancer	^82^

**Table 6:** Peptide–drug conjugates in clinical/preclinical trials for Food and Drug Administration approval.

PDC name (Manufacturer)	Sequence	Drug	Target	Target disease	Status
ANG1005 (AngioChem)	TFFYGGSRGKRNNFKTEEY (Angiopep-2)	Paclitaxel	LRP-1	Breast cancer with brain metastasis	Phase III
BT1718 (Bicycle Therapeutics)	-	Mertansine (DM1)	Membrane type 1-matrix metalloprotease	Esophageal tumor	Phase II
BT8009 (Bicycle Therapeutics)	CP(1Nal)dCM(hArg)DWSTP(HyP)WC	MMAE	Nectin-4	Metastatic non-small cell cancer	Phase I/II
CBX-12 (Cybrexa Therapeutics)	ACEQNPIYWARYADWLFTTPLLLLDLALLVDADEGTG (pHLIP®)	Exatecan	Low pH	Advanced solid tumors	Phase I/II
OPD5 (Oncopeptides AB)	-	Melflufen	Aminopeptidase	Relapsed multiple myeloma	Phase I
SBI-1301 (Soricimed Biopharma)	-	Paclitaxel	Transient receptor potential vanilloid subfamily member 6	Solid tumors	Preclinical
SG3299 (Spirogen)	NAVPNLRGDLQVLAQKVARTC	Tesirine	αvβ6 integrin	Pancreatic tumor	Preclinical
TH1902 (Theratechnologies)	GVRAKAGVRN(Nle)FKSESY	Docetaxel	Sortilin	Triple-negative breast cancer	Phase I

## Peptide inhibitors or antagonists of cell surface proteins

### Immune checkpoint inhibitors

**Fig. 3:**
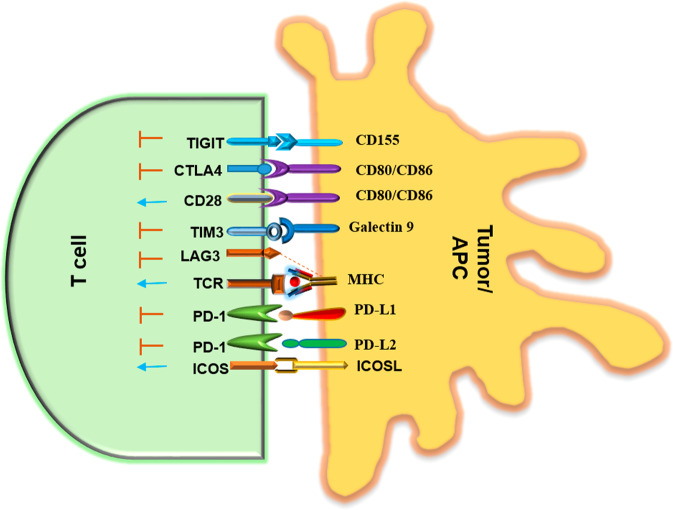
Types of immune checkpoints and their ligands. Interactions between immune checkpoints, such as CTLA-4 and PD-1 on T cells as well as CD80/CD86 and PD-L1 on antigen presenting and tumor cells, respectively, suppress T-cell activity. In addition, TIGIT, TIM3, and LAG3 on T cells play roles as immune checkpoints by interacting with their partners, such as CD155, galectin 9, and major histocompatibility complex, respectively. Blue arrows represent stimulatory signals, while red lines represent inhibitory signals.

The advent of immune checkpoint inhibitors (ICIs) has revolutionized the field of tumor therapy and promoted the development of more immune checkpoint blockades^83^. ICIs work by blocking the interactions between immune checkpoints such as CTL-associated protein 4 (CTLA-4), programmed cell death-1 (PD-1), and programmed cell death ligand-1 (PD-L1) and their ligands, which releases the inhibitory brakes of T cells and results in the robust activation of immune responses (Fig. 3). For example, CTLA-4, an inhibitory receptor expressed primarily by T cells, dampens T-cell activity and is upregulated upon T-cell activation^84,85^. At present, ICIs are used as first-line therapies for various solid tumors. Over the past decades, antibodies have been widely used ICIs. Ipilimumab was the first CTLA-4-blocking antibody approved by the US FDA for the treatment of human cancers. Anti-PD-1 antibodies such as pembrolizumab and nivolumab were included the second set of antibodies to be approved for the treatment of human malignancies, followed by anti-PD-L1 antibodies such as atezolizumab, durvalumab, and avelumab^86^.

PD-L1 is frequently upregulated in the tumor cell microenvironment as well as in DCs, macrophages, myeloid-derived suppressor cells (MDSCs), and regulatory T cells^87–90^. PD-L1 interacts with its ligand PD-1. Although T cells recognize tumor cells in the human body and kill them, the interaction between PD-1 on T cells and PD-L1 on tumor cells leads to T cell exhaustion^91–93^. Peptides that can block the PD-1/PD-L1 interaction and restore T cell activity against tumor cells have been identified^94–101^; these include peptide-57 (F(NMeAla)NPHLSWSW(NMeNle)(NMeNle)RCG), CLP001 (HYPERPHANQAS)/CLP002 (WHRSYYTWNLNT), and PD-L1Pep-1 (CLQKTPKQC)/PD-L1Pep-2 (CVRARTR) peptides. In addition to inducing T-cell reinvigoration through their PD-L1-blocking activity, PD-L1-binding peptides enable the targeted delivery of chemotherapeutic drugs to PD-L1-high tumors using PD-L1 as a tumor target. For example, PD-L1Pep-2 peptide-labeled doxorubicin-loaded liposomes increased the CD8 + T-cell/regulatory T-cell ratio in mouse colon tumor tissues more efficiently than combined treatment with PD-L1Pep-2 peptide and untargeted doxorubicin-loaded liposomes^101^. A prodrug nanoparticle synthesized by conjugating PD-L1Pep-2 with doxorubicin via cathepsin B-cleavable peptide linker (FRRG) inhibited tumor progression in the 4T1 mouse breast tumor model by inducing doxorubicin-mediated immunogenic cell death and blocking PD-L1 through PD-L1Pep-2^102^. Moreover, labeling peptides in nanoparticles increases the binding affinity of the peptide. For example, ferritin nanocages with multivalent PD-L1Pep-1 peptide bound to PD-L1 with a higher affinity than free PD-L1Pep-1 (~30 nM vs. 300 nM)^103^.

Recently, peptides that target next-generation immune checkpoints, such as T-cell immunoglobulin-3 (TIM-3), lymphocyte activation gene 3 (LAG-3), and T-cell immunoreceptor with Ig and ITIM domains (TIGIT), have attracted increasing attention. A TIM-3-binding peptide (GLIPLTTMHIGK) interferes with the binding of TIM-3 to Gal-9, the main ligand of TIM-3, thereby enhancing T-cell activity. Combining this peptide with a PD-L1 inhibitor exerted a tumor-suppressive effect in a mouse model^104^. A disulfide-bound cyclic LAG-3-binding peptide (CVPMTYRAC) interfered with the binding of LAG-3 to HLA-DR, the main ligand of LAG-3, activating CD8 + T cells while reducing the proportion of regulatory T cells^105^. A D-form version of a TIGIT-binding peptide (GGYTFHWHRLNP) identified from mirror-image phage display exhibited proteolytic resistance and prolonged half-life; it blocked the binding area of TIGIT to the poliovirus receptor (or CD155), enhanced the function of CD8 + T cells, and inhibited tumor growth^106^. The peptides that block immune checkpoints are summarized in Table 7.

**Table 7:** Peptides that inhibit immune checkpoints.

Name	Sequence	Target	Reference
−	NYSKPTDRQYHF	PD-L1	^94^
−	YASYHCWCWRDGRS	PD-L1	^95^
Peptide-57	F(NMeAla)NPHLSWSW(NMeNle)(NMeNle)RCG	PD-L1	^96^
−	GSGSGSTYLCGAISLAPKAQIKESL	PD-L1	^97^
CLP001, CLP002	HYPERPHANQAS, WHRSYYTWNLNT	PD-L1	^98^
−	SRLKEIANSPTQFWRMVARNTLGNGAKQSLNIEHARL	PD-L1	^99^
PD-L1Pep-1, PD-L1Pep-2	CLQKTPKQC, CVRARTR	PD-L1	^101^
YT-16	YRCMISYGGADYKCIT (cyclic)	PD-1	^100^
P26	GLIPLTTMHIGK	TIM-3	^104^
C25	CVPMTYRAC (cyclic)	LAG-3	^105^
−	GGYTFHWHRLNP	TIGIT	^106^

### Peptide antagonists of receptor tyrosine kinases, kinase-associated receptors, and other surface proteins

Tumor cells express abundant cell surface receptors for growth factors. Thus, receptor blockers or antagonistic antibodies and peptides can be used as anticancer agents. c-Met is a receptor tyrosine kinase that is overexpressed in numerous tumors. It binds to hepatocyte growth factor (HGF) and plays an important role in tumorigenesis and metastasis^107^. Using computer simulation, novel sequences of peptides, including the CM7 peptide (DQIIANN), have been designed to bind to c-Met with high affinity. This novel peptide bound to c-Met-expressing cells, inhibiting c-Met-mediated cell migration and invasion and tumor progression in mice^108^. A disulfide-constrained HGF-binding peptide, namely, HB10 (VNWVCFRDVGCDWVL), inhibits HGF–c-Met binding^109^. Soluble heparin-binding epidermal growth factor (sHB-EGF) is another target in combating cancer tumorigenesis and metastasis. Two sHB-EGF-binding peptides, namely, DRWVARDPASIF and TVGLPMTYYMHT, have been identified using phage display. They suppressed the activity of sHB-EGF to promote ovarian tumor cell migration and invasion by inhibiting the EGFR signaling pathway^110^.

CD44 is a cell surface receptor involved in cell adhesion to the extracellular matrix^111^. Although CD44 is expressed in normal cells, its alternative splicing isoforms, including CD44 variant 6 (CD44v6), are upregulated in tumor cells, contributing to tumor cell migration and metastasis by interacting with c-Met^111^. Using structural analysis, v6pep (KEQWFGNRWHEGYR) was selected from the human CD44v6 domain that interacts with c-Met and inhibits tumor growth and metastasis in a pancreatic cancer model^112,113^. Presently, v6pep is undergoing clinical trials. By screening a phage-displayed random peptide library, the NLN (CNLNTIDTC) and NEW (CNEWQKLSC) peptides that bind to CD44v6-expressing cells were selected; these peptides hindered HGF-mediated c-Met activation, thereby inhibiting CD44v6-high tumor cell migration and invasion^55^.

Certain tumor-derived exosomes contain heat shock protein 72 (Hsp72) in their membrane and interact with Toll-like receptor 2 (TLR2) on MDSCs, thereby activating cells^114^. The A8 peptide (SPWPRPTY) blocked the interaction between Hsp72 and TLR2 and the subsequent activation of MDSCs, thereby inhibiting tumor progression and potentiating the antitumor effect of chemotherapeutic agents, such as cisplatin^115^. Thus, peptides that act as cell surface protein antagonists are potential tools for inhibiting tumor progression and metastasis and can be administered alone or in combination with chemotherapy. The peptides that block cell surface receptors described here are summarized in Table 8.

**Table 8:** Peptides that serve as inhibitors or antagonists of cell surface proteins.

Name	Sequence	Target	Reference
CM7	DQIIANN	c-Met	^108^
HB10	DRWVARDPASIF, TVGLPMTYYMHT	sHB-EGF	^109,110^
v6pep	KEQWFGNRWHEGYR	CD44v6	^112,113^
NLN, NEW	CNLNTIDTC, CNEWQKLSC	CD44v6	^55^
A8 peptide	SPWPRPTY	Hsp72	^115^

### Peptide antagonists of hormone receptors

Some cancers depend on hormones to grow; thus, blocking the action of hormones can slow or control cancer growth. This kind of therapy is known as hormone therapy or endocrine therapy. At present, hormone therapy is applied to certain kinds of cancers, such as breast and prostate cancers. Hormone therapy, when used before surgery or radiation therapy as an adjuvant therapy, can decrease tumor size and lower the risk for tumor recurrence.

Gonadotrophin-releasing hormone (GnRH), also known as luteinizing hormone-releasing hormone, is released from the hypothalamus. It binds to a GnRH receptor in the pituitary to increase the production of follicle-stimulating and luteinizing hormones, thereby stimulating the release of estrogen by the ovaries^116^. When a GnRH analog is first administered, it produces a surge in ovarian hormones that can also cause several adverse effects, such as hot flashes. However, the long-term administration of the GnRH analog reduces ovarian hormone production and secretion, which downregulates and desensitizes the GnRH receptor in pituitary gonadotropic cells^116^. The GnRH receptor is also found in certain cancers, and the reduction in circulating estrogen slows the growth of hormone receptor-positive tumors such as ovarian cancer^117^, prostate cancer^118^, and breast cancer^119–121^. The use of GnRH analogs in clinical settings has been complicated because of their short half-life. However, with some modifications in its amino acids, long-lasting analogs have been successfully developed and used in the treatment of breast and prostate cancers. GnRH analogs that are currently used in clinics include goserelin (Zoladex®), (pGlu)HWSY(_D_-Ser(Bu^t^)LRP), leuprorelin or leuprolide (Lupron®, (pGlu)HWSY(_D_-Leu)LRP), and triptorelin (Decapeptyl®, (pGlu)HWSY(_D_-Trp)LRPG).

Somatostatin (AGCKNFFWKTFTSC) is a peptide produced by paracrine cells located throughout the gastrointestinal tract and binds to somatostatin receptors (SSTRs). Octreotide (FCFWKTCT) is a somatostatin analog that binds to SSTR2 and SSTR5 and serves as a growth hormone, insulin, and glucagon inhibitor^122^. Octreotide is used to treat severe diarrhea caused by certain intestinal tumors, such as vasoactive intestinal peptide-secreting tumors or metastatic carcinoid tumors.

## Peptide inhibitors of intracellular protein–protein interactions

Intracellular protein–protein interactions (PPIs) play a critical role in cells; for example, they facilitate the formation of protein complexes for signal transduction and facilitate the binding of transcription factors to promoters and enhancers. Thus, pharmacological approaches have been exploited to inhibit intracellular PPIs; related compounds include small molecules based on chemicals with a molecular weight <500 Da and biologicals based on proteins with a molecular weight >5000 Da. Small molecules efficiently cross the cell membrane, and they regulate the action of intracellular proteins^123^. However, these drugs cannot recognize a single mutation at the target site, and tumor cells easily acquire resistance against these drugs. In addition, the large surface of proteins involved in the interaction among proteins is not covered by small molecules because their sizes are too small^124^. In contrast, biologicals can bind to larger interfaces of proteins with high selectivity. However, they have poor cell permeability^125^. In addressing the limitations of small molecules and biologicals, peptides that interfere with PPIs with a molecular weight ranging between 500 and 5000 Da have been developed^126^. Peptides have the benefits of small molecules and biologicals, including the cell permeability of small molecules and the high selectivity of biologicals covering a large surface of proteins^127^. Considering that the sequence of peptide inhibitors frequently originates from endogenous proteins involved in the interaction, most of them serve as competitors of native protein interactions^128^.

c-Myc is a transcription factor involved in diverse human malignant tumors^129,130^. It usually forms heterodimeric complexes with its partner transcription factors to bind to DNA and regulate gene expression^131^. A peptide comprising 14 amino acids (RQIKIWFQNRRMKWKK) that originated from the helix 1 C-terminal region of Myc blocks the interaction between c-Myc and its partner^132,133^. Another example is OmoMyc, which comprises 92 amino acids and originates from the bHLHZip region of Myc but differs from Myc in four amino acid residues^134–136^.

Homeobox (HOX) is an important transcription factor for body segmentation and patterning during vertebrate development^137^. HOX gene expression is generally enhanced in tumors and is associated with angiogenesis, metastasis, and proliferation of tumor cells^138,139^. A common cofactor of HOX is preB-cell leukemia homeobox (PBX)^140,141^. The HXR9 peptide (WYPWMKKHHRRRRRRRRR) interferes with the interactions between HOX and PBX in several mouse tumor models^139,142^.

KRAS is an oncogenic protein that is commonly activated in many tumors, including lung cancer and pancreatic cancer, and it has been considered an undruggable target because it lacks a classical drug binding site^143,144^. The KRpep-2d peptide (Ac-RRRR-cyclo(CPLYISYDPVC)-NH2), a macrocyclic peptide that is a cyclic peptide containing >12 amino acids, and its derivatives bind to KRAS and inhibit KRAS-downstream signaling and cell proliferation^145,146^.

## Perspectives: improvement of the pharmacokinetic properties and biological activity of peptides

**Fig. 4:**
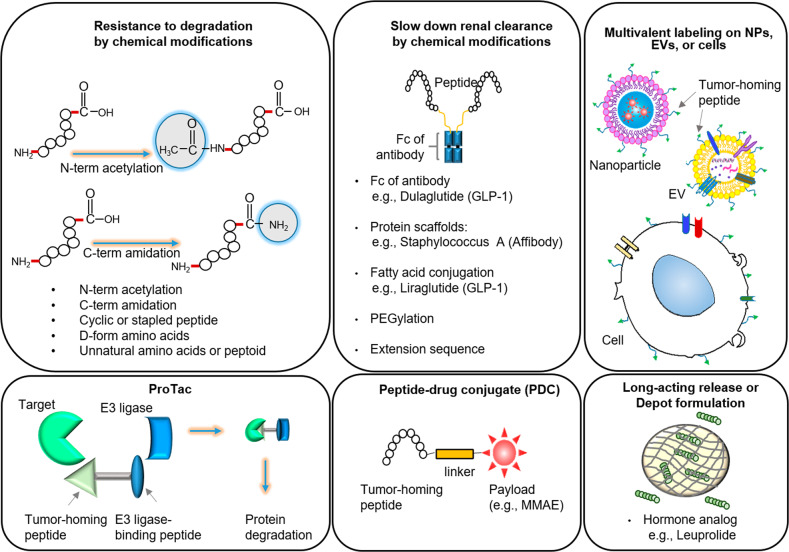
Diverse modifications of peptides to improve pharmacokinetic properties and enhance biological activity. Protease-mediated degradation and renal clearance of peptides can be reduced via chemical modifications. The long-acting release of peptides can be obtained by certain formulations, such as “depots”. Peptide-based ProTac, peptide–drug conjugates, and multivalent labeling on nanoparticles can improve the pharmacokinetic properties and biological activity of peptides.

Several approaches have been exploited to address or reduce the drawbacks of peptides as therapeutics (Fig. 4). First, to increase resistance to degradation, peptides are chemically modified through cyclization, which involves formation of disulfide bonds or formation of a stapled peptide; through N-term acetylation or C-term amidation; through modification to D-form amino acids; and through replacement of amino acids with unnatural amino acids or peptoids. Second, to slow down the excretion out of the body and increase half-life in the blood, peptides can be fused with the Fc fragment of an antibody and protein scaffolds such as *Staphylococcus* A antigen (Affibody) or conjugated with polyethylene glycol and fatty acids to enables the peptides to bind to albumin. Third, multivalent labeling of tumor-homing peptides on drug-loaded nanoparticles, EVs, and cells can enhance the binding activity and stability of peptides. Fourth, a peptide that binds to an intracellular target protein can be combined with a peptide that binds to an E3 ligase to degrade the target protein via proteolysis targeting chimera (ProTac) technology. Such peptide-based ProTacs have already been reported^147,148^. Fifth, tumor-homing peptides are linked with chemotherapeutic drugs to increase the antitumor activity of peptides. Finally, peptides are loaded into long-acting release microspheres or depots and injected into tissues to slowly release peptides for a longer time.

At present, a major portion of peptide therapeutics in the clinic are diabetes drugs such as liraglutide and dulaglutide. In the current market, peptide-based anticancer therapeutics include hormone analogs such as octreotide, leuprolide, and goserelin. In the future, an increasing number of peptide therapeutics will be developed in the field of cancer therapy; these could include tumor-homing peptides for targeted delivery of nanoparticles or EVs, peptide antagonists against cell surface proteins, and interference peptides against PPIs. In addition, PDCs could be used as an alternative to ADCs for certain cancers. Moreover, peptide-based ProTac technology will address the resistance of tumor cells to chemotherapy and will be a potential tool for cancer therapy.
